# Beyond Vision: Unveiling the Psychiatric Dimensions of Keratoconus

**DOI:** 10.3390/medicina61111943

**Published:** 2025-10-30

**Authors:** Teodor-Georgian Nuță, Mihnea Costin Manea, Corina Ioana Varlam, Gabriela Nuță, Aliss-Mădălina Mareș, Floris Petru Iliuță

**Affiliations:** 1 Department of Psychiatry and Psychology, Discipline of Psychiatry, Faculty of Dental Medicine, “Carol Davila” University of Medicine and Pharmacy, 010221 Bucharest, Romania; 2 Department of Adult Psychiatry, “Prof. Dr. Alexandru Obregia” Clinical Hospital of Psychiatry, 041914 Bucharest, Romania; 3 Department of Clinical Neurosciences, “Carol Davila” University of Medicine and Pharmacy, 050474 Bucharest, Romania; 4 Department of Neurology, Colentina Clinical Hospital of Bucharest, 020125 Bucharest, Romania

**Keywords:** keratoconus, depression, schizophrenia, ADHD, personality, psychiatric disorders, eye rubbing, vision loss

## Abstract

*Background and Objectives*: Keratoconus (KC) is a progressive corneal ectasia with multifactorial etiology, increasingly studied for potential associations with psychiatric disorders. This systematic review aimed to evaluate recent evidence linking KC with depression and other psychiatric conditions, including psychotic disorders, personality disorders, attention deficit hyperactivity disorder (ADHD), Tourette syndrome (TS), autism spectrum disorder (ASD), and obsessive–compulsive disorder (OCD). *Materials and Methods:* Following PRISMA guidelines, PubMed, ScienceDirect and SpringerLink were searched for English-language observational studies published since 2015 that examined psychiatric disorders in adults with keratoconus. We excluded reviews, case reports, pediatric, non-English, and inaccessible articles. Study quality was assessed using the Newcastle–Ottawa Scale and JBI Checklist. Data were narratively summarized and tabulated—without meta-analysis due to heterogeneity. *Results*: Twelve studies met inclusion criteria, including 41,906 KC patients and 63,267 controls. Eleven studies investigated depression and one ADHD. Findings on depression were mixed: five studies showed higher depressive symptoms among KC patients, while others found no significant association. Most were cross-sectional and of moderate-to-high quality. The single study on ADHD reported a higher prevalence of KC in males, but no evidence of casual association. Evidence on TS, ASD, and OCD was scarce and largely limited to case reports. The review was limited by heterogeneous methodologies, small sample sizes, an absence of longitudinal data, and reliance on self-report or registry data. *Conclusions*: Current evidence indicates increased psychological burden among some individuals with KC, particularly regarding depressive symptoms, yet casual relationships remain unproven. Male ADHD patients may have an elevated risk of KC, especially in the presence of eye rubbing. *Registration:* Not registered.

## 1. Introduction

Keratoconus (KC) is a progressive disorder of the cornea, involving both its thinning and ectasia. It is usually bilateral and asymmetric, also known to be associated with a plethora of other conditions [1]. KC most commonly manifests during adolescence and early adulthood, has a prevalence between 120 and 4790 per 100,000 individuals and is diagnosed more frequently in certain parts of the world. This condition affects both men and women, but the prevalence differences between the sexes are still disputed [2].

The understanding of its pathogenesis and etiology is still limited, but current knowledge is that KC is a complex disease influenced by a variety of both genetic and environmental factors involved in its development, caused by an abnormal response of the tissues to a number of stimuli which leads to keratocyte depletion or dysfunction, and loss of collagen, thus resulting in a weak cornea with a degenerated extracellular matrix. This phenomena involves simultaneous interplay between healing and destructive processes [3,4].

Despite the challenges of studying KC due to the absence of adequate animal models, the rapid degradation of human specimens and the lack of healthy human control groups, there are certain medical conditions that have been identified as predisposing individuals to developing KC [5]. Genetic defects that have been thought to increase one’s susceptibility to KC include Down syndrome, connective tissue disorders (including Ehlers Danlos syndrome, osteogenesis imperfecta and mitral valve prolapse) and Leber congenital amaurosis, even though this has been recently disputed in a review that considered the strength of the evidence inadequate with regard to the association with connective tissue disorders, and also suggesting that the link between KC and Down syndrome is still unclear possibly due to a lack of proper ways of diagnosing KC in the earlier studies [6,7,8]. Atopy-related conditions (allergic rhinitis, asthma, atopic dermatitis and ocular allergy) are significant environmental factors associated with the development of KC, especially since eye rubbing, a common behavior seen in patients with atopic disease, can cause or aggravate KC through mechanical and thermal damage to the cornea [5,6]. It has been proposed that in patients with psychiatric disorders that involve repetitive eye rubbing, this mechanism is of particular significance with regard to KC development and progression, ocular pruritus being considered a preventable risk factor [9].

The relationship between mental health and KC has been investigated more thoroughly in recent years. A number of papers have examined the possible link between KC and certain psychiatric conditions, such as mood disorders, psychotic disorders, attention deficit hyperactivity disorder (ADHD), Tourette syndrome (TS), autism spectrum disorder (ASD), and personality disorders [9,10,11,12,13]. These papers employed various methodologies and were conducted in different geographical locations, studying a variety of psychiatric disorders in relation to KC. Since vision impairment leads to worse mental health outcomes [14,15], our aim was to review this recent body of knowledge to find the possible connection between keratoconus and specific mental illnesses.

## 2. Materials and Methods

A comprehensive literature search was conducted across the databases PubMed, ScienceDirect and SpringerLink. Search filters were applied to restrict results to articles published in 2015 or later. The final search was performed on the 15th of September 2025. The search strategy combined the following keywords and Boolean operators: (“keratoconus”) AND (“psychiatric”) AND (“depression” OR “anxiety” OR “disorder” OR “ADHD” OR “Tourette” OR “schizophrenia” OR “autism” OR “personality”).

All identified records were imported into a reference management system, and duplicates were removed. Two reviewers independently screened the titles and abstracts of the deduplicated articles. This was followed by a full-text assessment of potentially eligible studies. Disagreements between the two reviewers were resolved by discussion with a third reviewer.

We deemed observational studies that investigated the association between KC and psychiatric disorders in adult human participants as eligible. Papers investigating pediatric populations, reviews, case reports, non-English studies and articles that we could not obtain full access to were excluded from our research. Additional publications were identified through manually searching through the reference lists of previously included articles. During the whole process, no automation tools were used. This resulted in the inclusion of 12 publications in our systematic review.

Data were extracted by one of the authors using a standardized form that included author, year of publication, the country the research was conducted in, study methodology, number of patients and controls, depression diagnostic method, main findings and limitations. The data were then independently reviewed by the other two authors. The primary outcomes were psychiatric conditions associated with keratoconus (depression, ADHD, psychotic disorders, personality disorders, TS, ASD, OCD). Data were extracted on all reported measures relevant to these domains (e.g., HDRS, BDI, PHQ-9, ZDS). When multiple instruments assessed the same domain within a single study, results from all relevant measures were included to ensure comprehensive synthesis. None of the studies evaluated depression at more than one point in time. Considering the fact that the majority (91.66%, *n* = 11) of the publications investigated the association between KC and depression, we decided to keep a systematic approach to our review with regard to depression only. For case–control and cohort studies, risk of bias assessment was conducted on these articles using the Newcastle–Ottawa scale which evaluates the selection of patients, the comparability of the patient groups and the ascertainment of exposure or outcome. Thus, studies were categorized as having either a low (≥7 stars), moderate (5–6 stars), or high risk of bias (≤4 stars), with a maximum quality score of nine stars. For cross-sectional studies, the JBI Checklist for analytical cross-sectional studies was used. In order to evaluate the quality of these papers, we decided to also make a quantitative assessment based on the answers to this checklist. We attributed 1 point for each “Yes” answer, 0 points for each “No” answer and 0.5 points for each “Unclear” answer. Article quality was described as high (score ≥ 7), moderate (score of 5–6) or low (score ≤ 4).

Due to the fact that only one paper (8.33%, *n* = 1) investigating the relationship between KC and psychiatric conditions (with the exception of depression) met our criteria, those associations were explored separately, using a less structured, narrative approach. This entailed a second process of screening through the deduplicated publications that we mentioned initially. We included articles that described the association between KC and psychotic disorders, ADHD, TS, ASD and personality disorders. We excluded reviews, non-English articles and not fully accessible papers. No automation tools were used during this process either.

We did not perform a meta-analysis, thus formal statistical tests for publication bias were not applicable. However, we qualitatively evaluated potential reporting bias by assessing whether included articles selectively reported significant outcomes and comparing the balance of positive and null findings across studies.

This systematic review was conducted and reported in accordance with the PRISMA 2020 guidelines. It was not prospectively registered in any public registry, and no protocol was prepared.

## 3. Results

**Figure 1:**
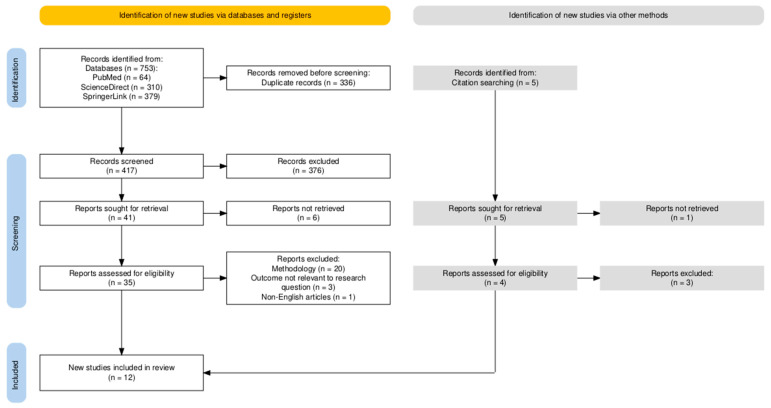
PRISMA flowchart.

Our initial search resulted in 753 publications (64 from PubMed, 310 from ScienceDirect and 379 from SpringerLink). A total of 336 duplicates were excluded and then the remaining 417 deduplicated publications were screened by reading their titles and abstracts. After retrieval, 35 papers were assessed for eligibility. Among the 35 reports evaluated, 20 were excluded because of their methodology (reviews, case series or case reports), three were excluded because their outcomes were not relevant to the study objective, specifically, they did not examine the association between keratoconus and psychiatric disorders, and one was excluded for not being published in the English language. The process of citation searching resulted in five articles that had the potential to be included; one of them could not be retrieved and lastly one of them met our criteria. Finally, 12 papers met both our inclusion and exclusion criteria. The majority (91.66%, *n* = 11) were investigating the relationship between KC and depression. One article (8.33%) studied the association between KC and ADHD. The PRISMA flowchart can be seen in Figure 1.

### 3.1. Depression and Keratoconus

The risk of bias assessment results can be found in Table 1 for six case–control or cohort studies. All studies obtained scores between 7 and 8, indicating a low risk of bias. Most demonstrated an adequate selection of participants, appropriate comparability between groups, and reliable ascertainment of exposure or outcome. Methodological limitations were noted primarily in outcome assessment and reporting completeness.

Table 2 presents the quality assessment for five cross-sectional studies. Methodological quality was more variable in this group. One study was rated as high quality, two as moderate to high, one as low to moderate, and one as low quality. The most frequent shortcomings of the included publications involved the lack of identification or adjustment for confounding factors, the absence of control groups, and incomplete reporting of sampling procedures.

**Table 1:** Risk of bias of case–control and cohort studies.

Authors	S1	S2	S3	S4	C	E1 or O1	E2 or O2	E3 or O3	Total	Risk of Bias
Marx-Gross et al. [16]	1	1	1	1	2	1	1	0	8	Low
Moschos et al. [17]	1	1	1	1	1	1	1	1	8	Low
Lin et al. [1]	0	1	1	1	2	0	1	1	7	Low
Aslan et al. [18]	1	1	1	1	1	1	1	0	7	Low
Bak-Nielsen et al. [19]	0	1	1	1	2	0	1	1	7	Low
Woodward et al. [20]	0	1	1	1	2	0	1	1	7	Low

Abbreviations: S1 = selection 1; S2 = selection 2; S3 = selection 3; S4 = selection 4; C = comparability; E1 = exposure 1; E2 = exposure 2; E3 = exposure 3; O1 = outcome 1; O2 = outcome 2; O3 = outcome 3.

We reviewed a total of 11 papers regarding the relationship between KC and depression. A summary of their main characteristics and outcomes can be found in Table 3. Depression was assessed using various standardized instruments, including PHQ-9 (Patient Health Questionnaire-9), BDI (Beck Depression Inventory), ZDS (Zung Self-rating Depression Scale) and HDRS (Hamilton Rating Scale for Depression), or through medical record data. The findings were heterogeneous across studies. Several investigations [17,18,19,21,22] reported significantly higher rates or severity of depressive symptoms among KC patients compared to controls, suggesting that KC may act as a risk factor for depression or worsen existing symptoms, particularly in patients with reduced visual acuity. In contrast, other studies [14,16,20,23,24] found no significant association between KC and depression.

Across the included papers, common limitations included small sample sizes, reliance on self-reported measures or retrospective medical records, insufficient control for disease severity or sociodemographic variables, and cross-sectional designs precluding causal inference. Taken together, the reviewed evidence indicates methodologically sound but inconsistent findings, thus no conclusive linkage between KC and depression was found.

**Table 2:** Risk of bias for cross-sectional studies.

Authors	1. Inclusion	2. Setting	3. Exposure	4. Criteria	5. Confounders Id.	6. Confounder Adj.	7. Outcomes	8. Analysis	9. Sample	10. Ethics	Quality
Yildiz et al. [21]	Yes	Yes	Yes	Yes	No	No	Yes	Yes	No	Yes	Moderate
Florek et al. [23]	Yes	Yes	Yes	Yes	Yes	No	Yes	No	Yes	Yes	Moderate-High
Al-Dairi et al. [22]	Yes	No	No	Unclear	Unclear	No	No	No	Yes	Yes	Low
Jonas et al. [24]	Yes	Yes	No	Yes	Yes	Yes	No	Yes	Yes	Yes	High
Alfardan et al. [25]	No	Yes	No	Unclear	Yes	No	Unclear	No	No	Yes	Low-Moderate

Abbreviations: Confounder Id. = confounding factor identification; Confounder Adj. = strategies to adjust for confounding factors.

**Table 3:** Main characteristics of studies investigating keratoconus and depression.

Authors	Publication Year	Country	Type of Study	Number of Patients/Controls	Depression Diagnostic Method	Main Result	Limitations of the Study
Lin et al. [1]	2021	Taiwan	case–control	5055/20220	Medical records	KC → ↓ depression	Reliance on medical records KC severity and visual parameters not reported
Bak-Nielsen et al. [19]	2019	Denmark	case–control	2679/26790	Medical records	KC diagnosis → ↑ depression risk	Reliance on medical records No KC severity data Long study period
Al-Dairi et al. [22]	2020	Saudi Arabia	cross-sectional	330	PHQ-9	40.6% of KC patients had depression	Sampling bias No clinical confirmation of depression Missing detailed clinical data No follow-up
Marx-Gross et al. [16]	2023	Germany	cohort study	12423	PHQ-9	KC ↔ depression	No KC severity data Possible selection bias
Jonas et al. [24]	2018	China	cross-sectional	3468	ZDS	Major ocular diseases ↔ depression	Self-reported depression Cultural influences on reporting No follow-up
Woodward et al. [20]	2016	USA	case–control	16053/16053	Medical records	KC ↔ depression	Reliance on medical records No data on the uninsured Lack of detailed clinical data
Alfardan et al. [25]	2023	Saudi Arabia	Cross-sectional	57	Medical records	KC patients → high prevalence of psychiatric illness	Small sample size Reliance on medical records No control group
Yildiz et al. [21]	2021	Turkey	Cross-sectional	94	BDI	KC → high rates of depression	Small sample size No follow up No control group Age restricted to young adults
Florek et al. [23]	2024	Poland	Cross-sectional	99/92	HDRS, BDI	KC ↔ depression severity	Gender disparity Control group health self-reported Only symptom severity measured, not psychiatric disorder prevalence No follow-up
Moschos et al. [17]	2018	Greece	case–control	56/47	ZDI, PHQ-9	KC ↑ depression levels ↓ Vision → ↑ depression KC → ↑ risk of depression	Small sample size No longitudinal follow-up Cannot assess effect of KC duration/severity
Aslan et al. [18]	2021	Turkey	case–control	59/65	BDI	KC → ↑ depression scores Vision and topographical parameters ↔ depression	Majority had mild KC Non-response rate unclear No adjustment for confounders

Abbreviations: N/A = not applicable; PHQ-9 = Patient Health Questionnaire-9; ZDS = Zung Self-rating Depression Scale; BDI = Beck Depression Inventory; HDRS = Hamilton Rating Scale for Depression; ↑ = increase; ↓ = decrease; → = associated with; ↔ = no significant association.

### 3.2. Psychotic Disorders and Keratoconus

The relationship between KC and psychotic disorders is definitely a much less explored subject relative to depression. This could be explained by the much lower prevalence of such disorders in KC patients, similar to that in the general population. One study found that 10.5% of patients with KC and a co-occurring psychiatric disorder were diagnosed with schizophrenia compared to 63.2% who were diagnosed with anxiety disorders and 56.1% who were diagnosed with depression [25]. One genetic study found no causal genetic association between KC and schizophrenia, suggesting that the association between KC and this mental disorder is due to different, environmental, mechanisms [13]. Additionally, two research papers propose that the possible association between KC and schizophrenia may be due to a common pathophysiological mechanism involving increased oxidative stress, which is present in both conditions [10,13].

We also mention three cases of patients with both KC and schizophrenia or schizoaffective disorder. The authors suggest that KC seems to occur after or at the same time as the initiation of antipsychotic medication [26]. This, in turn, implies that KC is being diagnosed after such a psychiatric illness has been identified.

### 3.3. Personality Disorders and Keratoconus

One study examining the personality traits of patients with KC revealed a significant correlation between KC and higher scores on the Maudsley Obsessive Compulsive Inventory (MOCI) subscales, except for the “doubting” subscale. The duration of KC was positively associated with the “checking” and “slowness” MOCI subscales. “Neuroticism” scores were also higher in the patient group on the Eysenck Personality Questionnaire-Revised Short Form [18]. Another study found that patients with KC had a nine-fold increased risk of cluster C personality disorders, more pronounced psychosomatic symptoms and a neurotic temperament [27].

Interestingly, Florek et al. found that the KC group exhibited a higher intensity of antisocial and schizotypal personality traits, though only women with KC exhibited more pronounced obsessive–compulsive traits [23].

Mark et al. concluded that there appears to be a connection between certain personality traits and chronic eye disease, but not specifically with KC [14].

### 3.4. Other Psychiatric Disorders and Keratoconus

During our research we found a paper presenting the case of a boy with a history of high-functioning autism spectrum disorder who also developed KC in both eyes. Notably, the patient also had a history of allergic rhinitis. After undergoing corneal cross-linking and behavioral modification intervention to break the habit of rubbing his eyes, his ocular disorder stopped progressing [28]. One cross-sectional study compared the corneal topography of ASD patients with typically developing controls and while this paper did not specifically aim to determine the prevalence or risk of KC, it suggested that there is no evidence of increased KC-like features in these patients [29].

One case report described a 27-year-old male with TS which manifested as various motor and vocal tics, who was also diagnosed with bilateral, asymmetric KC. Interestingly, the authors noted that the patient applied pressure to his eyeballs in order to prevent aggressive impulses. This behavior led to corneal perforation in his left eye [30]. Shinzawa et al. published a paper presenting three more male patients with TS who also had KC, and corneal cross linking was performed on all of them [9]. Notably, the authors reported that all patients had a habit of eye rubbing, which could have caused the progression of both KC and retinal damage. The latter was observed in two out of three patients and required surgical treatment [9].

A study explored the involvement of eye rubbing in KC and found that ocular pruritus was significantly more frequent in these patients. Eye rubbing was also significantly associated with skin pruritus and a history of allergies. However, there was no significant difference between the eye-rubbing and non-eye-rubbing groups regarding psychiatric or addictive histories. However, only 24.8% of the patients included in this study had been diagnosed with a psychiatric disorder, and an even smaller proportion (3.9%) had a follow-up evaluation [31].

With regard to ADHD and KC, one study was included in our systematic review. We assess this paper to be of high quality considering the fact that it performs well on the JBI Checklist because of its clear inclusion criteria, rigorous description of the methodology, proper measurement of exposure and outcomes, identification and consideration for confounding factors and appropriate use of statistical analysis. This study found that males with ADHD were significantly more likely to be diagnosed with KC than those without ADHD. However, no association was found between ADHD severity and KC severity, and the researchers could not investigate the chronological relationship between the two disorders. Another limitation of the study is the lack of data regarding eye rubbing, which could also be relevant. The authors found no link between KC and autism, obsessive–compulsive disorder (OCD) or anxiety disorder [11].

A case report has presented ADHD and KC together, describing a 49-year-old man who had a habit of rubbing his eyes, especially his right eye, since childhood. The patient reported using this behavior to cope with stress and improve concentration during learning and working tasks. However, he also noted a decrease in the frequency of this habit after receiving treatment for ADHD [32].

There is also one case report of rapid KC progression in an OCD patient with eye rubbing that also developed retinal detachment [33].

### 3.5. Summary of Findings: Psychiatric Disorders in Keratoconus (See Table 4)

Population: Adult patients with keratoconus.

Comparison: Individuals without keratoconus.

Setting: Observational studies (2015–2025).

Outcome: Psychiatric comorbidities (depression, ADHD, psychotic disorders, ASD, OCD, TS, personality disorders).

**Table 4:** Summary of findings.

Outcome	No. of Studies (Design)	Participants (KC/Control)	Main Findings	Certainty of Evidence (GRADE)	Comments
**Depression**	11 (6 case–control/cohort, 5 cross-sectional)	40373/63267	Mixed results: 5 studies showed ↑ depressive symptoms; 4 found no difference; 2 showed ↓ or no association	⊕⊕◯◯ Low	Heterogeneous methodologies; different tools used (PHQ-9, BDI, ZDS, HDRS); causal inference not possible;
**ADHD**	1 (cross-sectional)	1533	↑ prevalence of KC in males with ADHD ADHD severity **↔** KC severity	⊕⊕⊕◯ Moderate	Confounders controlled; lack of data on eye rubbing limits interpretation;
**Psychotic disorders**	1 (cross-sectional) + 3 case reports + 1 (genetic MR)	60	KC ↔ schizophrenia; rare co-occurrence;	⊕◯◯◯ Very low	Evidence from limited case reports; not generalizable;
**Personality disorders**	3 (cross-sectional)	188/187	↑ obsessive-compulsive and neurotic traits ↑ risk of cluster C personality disorders	⊕⊕◯◯ Low	May reflect chronic disease burden rather than KC itself;
**TS**	2 (case reports + case series)	4	KC linked to compulsive eye rubbing	⊕◯◯◯ Very low	Based on case reports; no comparative data;
**ASD**	2 (1 case report, 1 cross-sectional)	115	No increased prevalence of KC-like features in ASD; isolated co-occurrence described;	⊕◯◯◯ Very low	Sparse evidence; not statistically assessed;
**OCD**	1 (case report)	1	Rapid KC progression associated with compulsive eye rubbing	⊕◯◯◯ Very low	Anecdotal evidence;

Abbreviations: KC = keratoconus; MR = mendelian randomization; ADHD = attention deficit hyperactivity disorder; TS = Tourette syndrome; ASD = autism spectrum disorder; OCD = obsessive–compulsive disorder; ↑ = increased; ↔ = no significant association; ↓ = decreased.

## 4. Discussion

Research investigating the potential link between KC and depression yielded mixed results, likely due to the variety of methodologies, populations and diagnostic methods used. Each study had its respective strengths and limitations. Therefore, we evaluate the reviewed research to be inconclusive regarding the common mechanisms involved in the development of these disorders. We consider that current knowledge does not highlight a direct correlation between KC and depression mainly because given the current data set, it is not feasible to evaluate whether or not KC is the primary driver of depression in these patients. Taken together, the available evidence supports the presence of increased psychological burden among some individuals with KC, yet does not conclusively establish a causal or universal linkage between KC and clinical depression. However, this particular subject could be a promising avenue for future research, especially for large, multicenter, longitudinal studies, that could clarify whether KC contributes causally to psychiatric disorders and better describe the temporal relationship between visual impairment and mental health outcomes. This research should use standardized psychiatric assessments rather than self-report scales whilst also taking into account KC severity and progression in order to assess how disease burden corelates with psychological and psychiatric outcomes.

A 2024 meta-analysis pooled available data and demonstrated that KC patients had significantly higher mean depression scores compared with controls, yet the pooled odds ratio for clinically diagnosed depression showed that this was not the case. The authors emphasized considerable heterogeneity and sensitivity to individual large studies, particularly those using registry data and variable index times [12]. These results indicate that while depressive symptomatology appears more common in KC, the true prevalence of diagnosed depressive disorders may not differ substantially from that of the general population. This distinction between symptom severity and diagnostic prevalence mirrors the mixed findings of the studies included in the present review. Despite the fact that in their review, Mark et al. focus their attention on the relationship between certain personality traits and KC, they also conclude that there is a bidirectional association between visual dysfunction and psychopathology, impaired vision potentially leading to worse mental health and vice versa [14]. This is significant with regard to KC as well, especially since it is a progressive disorder. Additionally, there is research showing that the reverse is also true: patients with depressive or anxiety symptoms have a higher likelihood of experiencing and reporting visual symptoms in the future [13,34]. In this case, depression has the potential to delay the diagnosis of a disorder responsible for visual impairment and lead to poor adherence to the treatment required for the eye disorder. Recently, it has been proposed that vision loss could be considered a psychosomatic disorder, Sabel et al. suggesting that vision loss reduces the subjective quality of life due to depression and anxiety and therefore low vision resulting in stress, stress that in turn could be a cause of different visual disorders and thus leading to a downward spiral [35]. However, none of these studies have explored the relationship between anxiety, depressive symptoms, and vision loss specifically in KC patients, making it challenging to determine whether their conclusions can be applied to this group.

Schizophrenia is a complex condition that is characterized by several brain anomalies; however, a recent review emphasized the importance of associated ocular disorders in these patients, which can be both structural and physiological [36,37,38,39,40]. The relationship between KC and psychotic disorders, particularly schizophrenia, is an understudied topic. After reviewing the existing body of literature, we conclude that there is not sufficient evidence to support a connection between KC and psychotic disorders. Such a relationship, if existent at all, is likely complex, multifactorial, and warrants further investigation.

Keratoconus appears to be associated with elevated obsessive–compulsive tendencies, neuroticism, and certain personality disorder traits, though some evidence suggests these features may reflect chronic eye disease in general rather than KC specifically.

Our findings suggest a link between ADHD and KC, particularly in males who exhibit compulsive eye rubbing. However, research on ocular disorders in patients with OCD, TS and ASD is insufficient to demonstrate a connection between these conditions and KC. This is probably due to the low prevalence of such disorders compared to depression, for example, which makes conducting research more challenging given the difficulty of gathering the necessary number of patients [11,25].

This study takes a broader look at the relationship between keratoconus and psychiatric disorders. Our research fills a critical gap in the literature by moving beyond the narrow focus of earlier work, which was almost solely preoccupied with depression. Our approach captures a wider spectrum of potential associations and by limiting our review to articles published in the past decade, we ensured that our findings are grounded in the most current diagnostic methods, criteria, and clinical practices, thereby enhancing their relevance to contemporary healthcare settings.

We also have to acknowledge several limitations of our review and the existing evidence base. One of our concerns was selection bias due to our decision to restrict this review to publications of the past 10 years only, but also because some studies have included patients with less severe KC, within specific age ranges, limited to certain populations, and one study used online recruitment. Moreover, positive studies are more likely to be published, whereas large studies with null results may remain unpublished, potentially exaggerating the observed associations. Second, variations in study design, sample size, and lack of a consistent characterization of depression led to inconsistent findings across the available studies which made comparing and synthesizing the results difficult. This was one of the main considerations when deciding against performing meta-analysis, which would have been more robust if a large number of methodologically similar studies were available. Third, the cross-sectional nature of many of the included studies limited our ability to determine causality. Fourth, many studies lacked detailed clinical data on KC severity, other systemic comorbidities and had no follow up evaluation. Additionally, regarding conditions that are less frequently diagnosed, we could only rely on case reports or small cohorts, limiting generalizability. Moreover, the overrepresentation of small studies reporting significant associations suggests possible publication bias favoring positive findings. Most small, cross-sectional studies reported significant associations between keratoconus and depressive symptoms, whereas larger registry-based studies tended to find none. This pattern may indicate publication bias favoring positive findings.

The pathophysiological overlap between keratoconus and psychiatric symptoms can be conceptualized within a biopsychosocial framework. At the biological level, KC is characterized by oxidative damage and chronic pro-inflammatory activity (elevated IL-6, TNF-α and MMPs) that mediate extracellular matrix remodeling and stromal weakening. These processes are associated with systemic oxidative–inflammatory signaling pathways that have also been implicated in depression and other psychiatric disorders. Behavioral drivers such as repetitive eye rubbing produce mechanical stress and local inflammation, providing a direct behavioral bridge between certain psychiatric traits (impulsivity, compulsivity, tics) and KC progression [41,42,43,44].

At the psychosocial level, progressive visual impairment and the chronic healthcare burden of KC can precipitate loss of valued activities, reduced participation and perceived stigma, all recognized risk factors for depression and anxiety in visually impaired populations. The relationship is likely bidirectional: psychiatric symptoms may increase risk behaviors (eye rubbing, treatment non-adherence) that worsen ocular outcomes, while worsening vision and treatment burden may exacerbate psychiatric morbidity [34,45]. Future studies should therefore simultaneously assess ocular biomarkers (oxidative/inflammatory markers), objective visual function and standardized psychiatric measures to disentangle causality and identify potential targets for integrated interventions.

We consider the co-occurrence of KC and psychiatric disorders to be particularly important given that people with visual impairments are at risk of poor mental health outcomes [15]. Since KC could have a prevalence as high as 5% in certain populations [2], ophthalmologists and psychiatrists should adopt a bidirectional, collaborative approach when caring for patients with KC. Ophthalmologists can play a key role in the early detection of psychological distress by incorporating brief mental health screening tools (e.g., PHQ-9, GAD-7) into assessments of patients showing depressive symptoms, anxiety, or maladaptive behaviors such as compulsive eye rubbing. Psychiatrists, on the other hand, should remain alert to visual complaints, excessive eye touching, or unexplained vision changes in their patients, prompting ophthalmologic evaluation when appropriate.

Regular interdisciplinary communication through shared medical records or joint case discussions can ensure that both visual and mental health aspects are addressed simultaneously. Collaborative psychoeducation and behavioral interventions can reduce harmful habits, improve treatment adherence, and enhance overall quality of life. This integrated model not only supports the early recognition of comorbidities but also promotes more comprehensive, patient-centered care.

## 5. Conclusions

This review of the most recent literature found increased psychological burden among some KC patients, yet a casual or universal linkage between KC and clinical depression cannot be established. Male patients with ADHD are more likely to develop keratoconus, but no causal connection was found. The co-occurrence of keratoconus with psychotic disorders, Tourette syndrome, autism spectrum disorder, or obsessive–compulsive disorder is rare, appearing only in a limited number of case reports. Keratoconus may be linked to obsessive–compulsive traits, neuroticism, and personality disorders, but these traits may actually be a result of chronic eye disease more generally.

## Data Availability

Information on data collection forms, extracted data, and other review materials is available from the corresponding authors upon reasonable request.

## Abbreviations

The following abbreviations are used in this manuscript:

KC	Keratoconus
ADHD	Attention deficit hyperactivity disorder
TS	Tourette syndrome
ASD	Autism spectrum disorder
OCD	Obsessive–compulsive disorder
PHQ-9	Patient Health Questionnaire-9
ZDS	Zung Self-rating Depression Scale
BDI	Beck Depression Inventory
HDRS	Hamilton Rating Scale for Depression
MOCI	Maudsley Obsessive Compulsive Inventory
MR	Mendelian randomization
IL-6	Interleukin 6
TNF-α	Tumor Necrosis Factor—alpha
MMPs	Matrix Metalloproteinases
